# Membrane for Pressure-Driven Separation Prepared with a Method of 3D Printing: Performance in Concentrating Orange Peel Extract

**DOI:** 10.3390/membranes15040105

**Published:** 2025-04-01

**Authors:** Priscila Pini Pereira, Isabela Pacola Gonçalves, Luiza C. A. Molina, Roberta Delcolle, Yuliya S. Dzyazko, Carolina Moser Paraiso, Guilherme L. Batista Neto, Alexandre Diório, Angélica Marquetotti Salcedo Vieira, Rosângela Bergamasco

**Affiliations:** 1 Department of Chemical Engineering, State University of Maringá, 5790 Colombo Ave., Maringá 87020-900, PR, Brazil; 2 V.I. Vernadskii Institute of General and Inorganic Chemistry of the National Academy of Science of Ukraine, Palladin Ave. 32/34, 03142 Kyiv, Ukraine; 3 Department of Food Engineering, State University of Maringá, 5790 Colombo Ave., Maringá 87020-900, PR, Brazil; 4 Department of Chemistry, State University of Maringá, 5790 Colombo Ave., Maringá 87020-900, PR, Brazil

**Keywords:** polylactic acid, sucrose, 3D-printed membrane, fused deposition modeling, ultrafiltration, orange peel extracts, polyphenols

## Abstract

3D-printing enables the fabrication of membranes with desired shapes and geometrical parameters. In this study, a membrane for pressure-driven processes was manufactured in a single step using the fused deposition modeling (FDM) technique. The membrane was produced from a mixture of polylactic acid (PLA) with sucrose as a pore-forming agent. Sucrose was removed from the final membrane by washing it with water. The membrane consists of three layers, and this sandwich-like structure ensures its mechanical stability. The material obtained was characterized using SEM and AFM imaging, as well as nitrogen adsorption-desorption and contact angle measurements. The porosity of each layer of the membrane is due to a loose region, which is coated on both sides with a dense film formed during printing. The pores responsible for rejection capability can be found in grooves between the polymer stripes in the dense layer. The membrane exhibits a water permeability of 64 L m^−2^h^−1^bar^−1^, with a molecular weight cut-off of 69 kDa. The PLA membrane can be used for polyphenol concentration, demonstrating a permeability of 2–3.4 L m^−2^h^−1^bar^−1^ and a selectivity towards these compounds of 78–98% at 0.5 bar, with a flux decline ratio of up to 50%.

## 1. Introduction

3D-printing was invented in the early 1970s [1]. The first patent was directed at an apparatus for the manufacture of three-dimensional products from polymers under the influence of laser radiation. Since then, 3D-printing has developed rapidly and intensively because it allows any consumer to create objects of the desired shape and size [1,2]. Other advantages of 3D-printing include time and cost efficiency, low energy consumption, and minimal residue generation. Today, 3D-printing is used both for military applications (such as ammunition components, communication devices, drones, and even jets) [3] and civilian needs [2], including medicine [4], machinery [5], fashion [6], and the food industries [7].

Recently materials produced by 3D-printing have been applied to membrane processes, particularly for water and wastewater treatment [8,9,10,11]. These materials are mainly spacers, components for membrane modules, adsorbents, catalysts, photocatalysts, and bio-carriers [9]. At the same time, 3D-printed membranes have also been developed [8,9,10,11]. Their potential application includes oil-water separation [12,13], water desalination [14,15], and the removal of toxic inorganic ions and organic contaminants [16,17,18]. Processes utilizing these materials include membrane distillation [19], gas separation [20], electro-membrane separation (anion exchange [21], cation exchange [22]) and mosaic membranes [23]), hemodialysis [24], nanofiltration [16] and reverse osmosis [24]. However, the production of ultrafiltration membranes remains limited by the low resolution of 3D-printing [25].

As a rule, the preparation of materials for pressure-driven separation involves more than one stage except in [12] (where the membrane pores are too large to provide the retention of small species). A thin active layer can be printed and attached to a macroporous substrate, which is prepared using conventional methods [14,26]. Another approach involves printing the substrate first, followed by coating it with an active layer using traditional techniques [13,27]. Thus, a key challenge is the one-stage fabrication of membranes capable of retaining colloidal particles.

To address this issue, the fused deposition modeling (FDM) method appears to be an attractive solution [8,9,10,11,28]. The method involves melting a polymer filament, followed by the extrusion of the molten material. The filament passes through a heated metal cylinder with a nozzle at the end. The molten mass leaves the nozzle, forming a stripe whose size corresponds to the nozzle diameter. The product is printed layer by layer, allowing the fabrication of objects in various shapes. FDM printers are widely available and use low-cost polymers as ink. However, the surface of printed products contains grooves at the junctions between stripes, making it less smooth. At the same time, this limitation of the FDM technique can be an advantage for printing porous membranes, since pores can be formed within these grooves.

The porosity of membranes is supposed to increase with the addition of a pore pore-forming agent to the printer ink. A well-known pore-forming substrate for this purpose is saccharose, which is used for pore formation in both ceramics [29,30] and polymers [31,32].

The aim of this study is to develop a one-stage 3D-printing method for fabricating porous membranes using a polymer containing a pore-forming agent and to evaluate its separation ability.

Since ultrafiltration is widely used in the food industry [33,34,35], the potential practical application of 3D-printed membranes in this field was also explored through the filtration of orange peel extract. It is well known that citrus fruits contain polyphenols such as hesperidin, narirutin, naringin, and eriocitrin, which possess antioxidant properties [36,37,38]. Citrus polyphenols reduce the risk of coronary heart disease and exhibit anti-carcinogenic and anti-inflammatory activity by acting as radical scavengers. Additionally, the antioxidant and antibacterial properties of citrus peel extracts have been reported [37,38]. For this reason, plant extract components are used not only for food and beverage production [39,40] but also in the pharmaceutical industry [41,42].

Since membrane separation is a non-destructive process, it is preferable for the recovery of polyphenols from plant extracts over conventional techniques [43]. Among various methods for polyphenol concentration, such as membrane distillation [44] or electrodialysis [45], pressure-driven processes are the most attractive [43] due to their low operating and material costs. Moreover, filtration does not cause phase transformation or variation in liquid acidity.

Since most polyphenols found in orange peels are poorly soluble in water, they exist in the form of nanoparticles in aqueous media [46]. Thus, they can be retained by ultrafiltration at a pressure of 3–6 bar, although the retention is not too high (up to 62–68%) using the membranes of 30–50 kDa [47]. Lower retention (up to 13–25%) is reported for microfiltration membranes with a pore size of 0.22–0.45 μm. Nanofiltration and reverse osmosis allow one to obtain higher retention (up to 90 and 98% respectively). The integrated process of polyphenol recovery from orange peel extracts has been developed, it involves micro-, ultra-, nanofiltration, and also reverse osmosis. In all cases, no considerable decrease in the permeate flux was found, this parameter shows insufficient fluctuations over time. The data [47] are suggested by [48] when various nanofiltration membranes were used. Rather high recovery of polyphenols (85–90%) is achieved at 6 bar [48].

It is reasonable to expect that polyphenols could be retained with a 3D-printed membrane under lower pressure if they exhibit ultrafiltration properties.

Thus, the objective of this study involves not only testing the membrane with water and calibrating solutions but also with orange peel extract.

## 2. Materials and Methods

### 2.1. Materials

The powder of polylactic acid (PLA) was purchased from Zhejiang Flashforge 3D Technology Co. Ltd., Hangzhou, People’s Republic of China). Orange residues were donated by the juice processing industry (Prats Company, Brazil). Bovine serum albumin (BSA), saccharose and materials for polyphenol analysis: gallic acid (C_7_H_6_O_5_), Coomassie Brilliant Blue G-250, phosphate-buffered saline, Folin-Ciocalteu reagent (a mixture of phosphomolybdate and phosphotungstate) and sodium carbonate (Na_2_CO_3_) were purchased from the Merck Group.

### 2.2. BSA Solution

The calibrating solution contained 1 g L^−1^ of BSA. This solution was also applied to the preparation of a series of standards for the photometric analysis of this protein (50–1000 mg L^−1^). Analysis was carried out with the Bradford method [49]. Briefly: a solution of Coomassie Brilliant Blue G-250 (100 mg in 50 mL of 95% ethanol) was prepared, followed by the addition of 100 mL of 85% phosphoric acid (H_3_PO_4_). The solution was then diluted with water to a final volume of 1 l, obtaining the protein reagent. Next, 0.1 mL of a solution containing protein was placed in test tubes and diluted with phosphate saline buffer up to 0.1 mL (the buffer pH was 7.4, it contained 0.162 mol L^−1^ Na_2_HPO_4_ and 0.038 mol L^−1^ NaH_2_PO_4_, the total phosphorus concentration was 0.2 mol L^−1^). Then, 5 mL of the protein reagent was added, and the absorption measurements were performed after 2 min at 595 nm using a UV-1800 spectrophotometer (Shimadzu, Kyoto, Japan). A blank solution was prepared using 0.1 mL of the phosphate buffer and 5 mL of protein reagent.

### 2.3. Preparation and Analysis of Orange Peel Extract

Orange residues were dried in an oven with air circulation at 50 °C for 48 h. After drying, they were crushed, and a particle fraction of 0.420–0.595 mm was selected using Tyler sieves. The resulting powder was mixed with distilled water was subjected to ultrasonic activation in an Ultracleaner 1650 ultrasonic bath (Unique, Curitiba, Parana, Brazil) at 40 kHz and 60 °C. The solid-to-liquid mass ratio was 1:100 (solution I) and 1:25 (solution II). Notably, at the ratio of 1:100, the extraction is completed within 1–2 min [50], preventing the chemical transformation of polyphenols under elevated temperatures [51]. Conversely, the maximal polyphenol yield is achieved at the ratio of 1:25 [50]. The mixture was then centrifuged at 8000 rpm for 20 min and filtered through vacuum filter paper to remove coarse particles [52]. The particle size distribution in the orange peel extract was analyzed using a ZETASIZER Nano Series ZSP analyzer (Malvern Instruments, Malvern, UK).

To determine the dry matter content, a 10 mL sample was evaporated at 100 °C in an oven under ambient pressure and air circulation [53]. The polyphenol content was analyzed using a spectrophotometric method [54]. Briefly, 0.5 mL of sample or standard (gallic acid) was taken, followed by the addition of 2.4 mL of distilled water, 2 mL of a 2% Na_2_CO_3_ solution, and 0.1 mL of Folin–Ciocalteau reagent. After incubation of the reaction mixture for 60 min under ambient conditions, the absorbance was measured at 750 nm against a water blank.

### 2.4. Ink for 3d Printer and Printing Membrane

To fabricate the porous membrane, solid sucrose (10 g) was mixed with PLA powder (50 g). A solvent containing 100 mL of acetone and 50 mL of chloroform was then added. After the complete dissolution of the solids, the mixture was kept at 65 °C for 90 min to allow the solvent evaporation. The resulting solid residue was crushed using a mill, followed by extrusion to produce filaments. These filaments were subsequently crushed into flat particles of irregular shape, which were then fed into a Piocreat G5 pellet 3D printer (Shenzhen Piocreat 3D Technology Co. Ltd., Shenzhen, People’s Republic of China) to manufacture the membranes from polylactic acid (PLA membrane).

**Figure 1:**
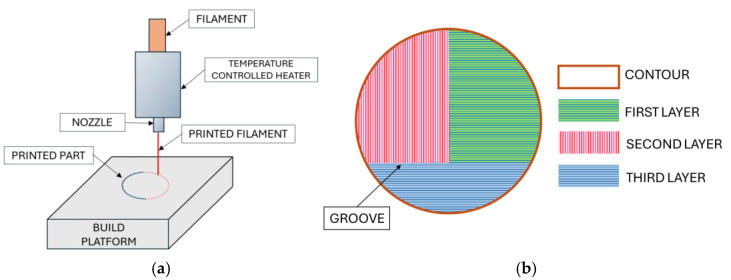
(**a**) Scheme of 3D-printing with FDM technique; (**b**) design of the three-layered membrane.

The membrane prototype was designed using Autodesk Fusion 360© (2020 Autodesk Inc., San Francisco, CA, USA). The model consisted of a three-layered cylinder structure. Printing was performed with a nozzle temperature of 210 °C, a print bed temperature of 60 °C, and an extruder nozzle diameter of 0.4 mm. A schematic representation of the 3D-printing process is shown in Figure 1a.

Slicing was performed using the printer’s dedicated software. To ensure smooth membrane edges, the outer perimeter was printed first as a continuous contour. Inside the contour, straight stripes were printed at a speed of 30 mm s^−1^, with no spacing between them. Three layers were printed to provide mechanical stability to the membrane, with adjacent layers oriented 90 ° to each other (Figure 1b). The FDM method inherently creates grooves at the junction of adjacent stripes, contributing to the membrane’s final porosity and texture.

### 2.5. Investigations of Membranes

Membrane imaging was performed by means of a scanning electron microscope (SEM Quanta 250 FEI Company, Hillsboro, OR, USA) under standard vacuum conditions. Preliminarily, the samples were fixed on metallic stubs using double-sided conductive tape, then the samples were coated with an ultrathin gold layer using a BAL-TEC Sample Coater (BAL-TEC, Los Angeles, CA, USA). Both the membrane surface and ground particles—immediately after the deposition from the mixed solvent—were analyzed. The accelerating voltage was 15 kV.

To further characterize the membrane surface, an atomic force microscope (AFM, Shimadzu SPM-9700, Kyoto, Japan) was employed. AFM images were obtained in dynamic mode with a silicon tip with a spring constant of 0.5–9.5 N m^−1^ and a resonance frequency of 4.5–9.5 kHz. The magnitudes of the root-mean-squared roughness (Rq) for the samples surface were estimated from 10 × 10 μm scans using the Gwyddion software 2020 (Gwyddion, Brno, Czech Republic).

The membrane thickness was determined using an MDC-25MX digital micrometer (Qualitec, Sao Paulo, Brazil).

Prior to nitrogen adsorption-desorption analysis, the membrane was ground in a ball mill and subsequently heated at 100 °C for 2 h. to remove residual moisture The measurements were carried out by means of a Micromeritics ASAP 2020 analyzer (Micromeritics Instrument Co., Norcross, GA, USA).

The contact angle (wetting angle) was measured with an OCA 15 PLUS goniometer (DataPhysicsInstruments GmbH, Filderstadt, Baden-Württemberg, Germany). Three drops of deionized water were carefully deposited at different locations on the membrane surface using a micro-syringe. The mean value of the wetting angles was determined.

### 2.6. Water Filtration

**Figure 2:**
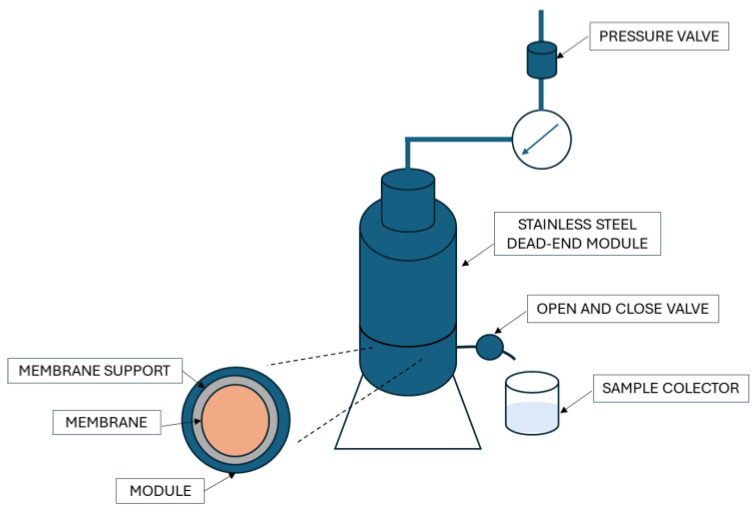
Scheme of the filtration system.

All filtration experiments were conducted using a filtration system developed at the State University of Maringá (Maringa, Parana, Brazil) (Figure 2). The system utilizes a dead-end filtration module with an effective membrane area (*A*) of 9.1 × 10^−4^ m^2^. The pressure was supplied by a compressor using atmospheric air, operating within a range of 0.5 to 2.0 bar. Filtration tests with water were carried out at 25 ± 2 °C.

Initially, distilled water was filtered at 0.5 bar, and the effluent was analyzed using UV-vis spectrophotometry. The UV spectra were recorded within the 200–400 nm range with a UV-1800 spectrophotometer (Shimadzu, Japan). Filtration was considered complete when the absorbance peak at 260 nm attributed to saccharose, disappeared. At this point, the membrane was dried at 50 °C until constant mass.

Subsequently, water filtration tests were performed at 0.5–2 bar, and the effluent volume (*V*) was recorded over time (*τ*) [55]:

(1) $$J=\frac{dV}{d\tau }\frac{1}{A}$$

where *J* is the flux, determined under steady-state conditions when the flux remained constant.

Immediately after the test, the membrane was weighed, and its porosity was determined using the following equation:

(2) $$\varepsilon =\frac{m_{w}-m}{\rho V_{m}}$$

where *m_W_* and *m* are the mass of wet and dry membrane respectively, *V_m_* is the volume of the wet membrane, and *ρ* is the density of water.

### 2.7. Filtration of Bsa Solution and Orange Peel Extract

Before the filtration of liquids containing the components of biological origin, distilled water was passed through the membrane, and the *J*_1_ flux was determined. Then, the BSA solution or orange peel extract was filtered, and the flux of the permeate was recorded as *J*_2_. Filtration was carried out at 25 ± 1 °C for BSA and 11 ± 1 °C for extract. The retention (*φ*) was calculated using [55]:

(3) $$\varphi =\frac{C_{0}-C}{C_{0}}\times 100\%$$

where *C*_0_ and *C* are the concentrations of feeding solution and permeate, respectively. Then, water was filtered again, and the flux *J*_3_ was determined. The following parameter was then calculated: the water flux recovery ratio (*FRR*) [56]:

(4) $$FRR=\frac{J_{3}}{J_{1}}\times 100\%$$

and flux decline ratio (*FDR*):

(5) $$FDR=\left(1-\frac{J_{2}}{J_{1}}\right)\times 100\%$$

were calculated. Reversible fouling, *R_r_*, was estimated according to the expression:

(6) $$R_{r}=FRR-(100\%-FDR)$$

Irreversible fouling, *R_ir_*, was determined as:

(7) $$R_{ir}=100\%-FRR$$

The *R_r_* and *R_ir_* parameters are the constituents of the *FDR* value:

(8) $$FDR=R_{r}+R_{ir}$$

The series of filtration experiments was carried out at 0.5, 1.0, 1.5 and 2 bar. The feed solution volume was 200 mL. However, when solution II, containing orange peel extract, was concentrated, a smaller volume (20 mL) was used. After each filtration series, the membrane was washed with NaOH and HCl (0.1 mol L^−1^ of each), followed by water (BSA). HCl and water were used for washing after the extract filtration similar to [57]. Additionally, the membrane was rinsed with ethanol.

## 3. Results

### 3.1. Membrane Characterization

**Figure 3:**
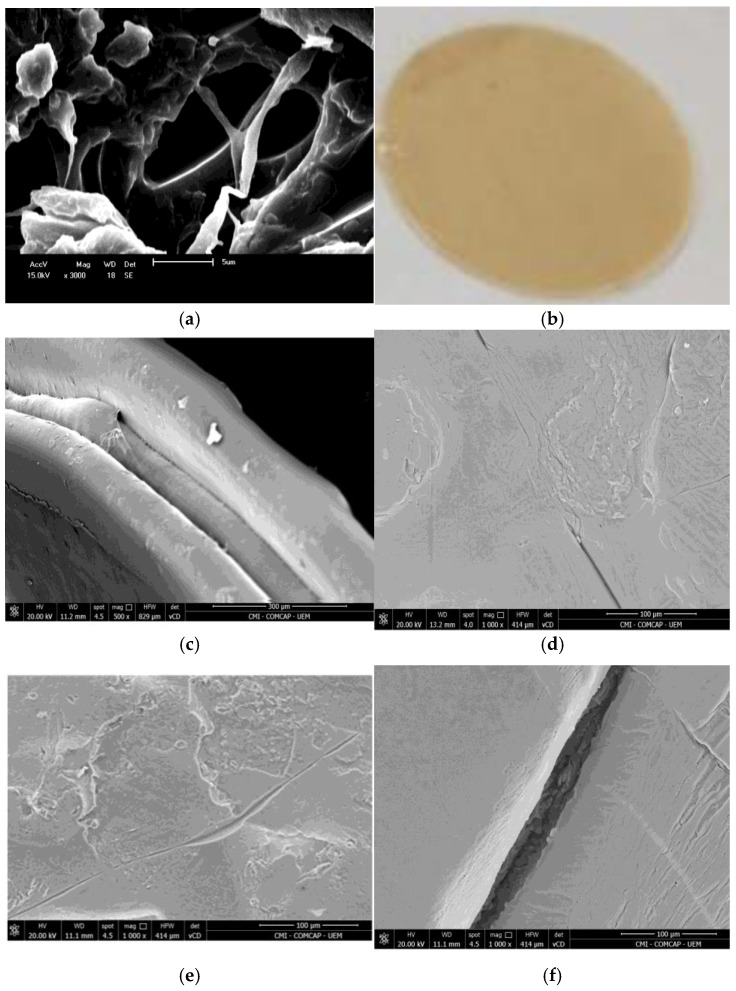
(**a**) SEM image of the PLA powder obtained from the sucrose-containing solution; (**b**) photo of 3D-printed membrane, (**c**) SEM images of the membrane: side-view, (**d**) face side, (**e**) bottom side, (**f**) chip of the membrane.

Dissolution of PLA in the mixed organic solvent containing sucrose results in the formation of a porous polymer with fibrous morphology (Figure 3a). The thickness of the fibers is uneven, ranging from 0.5 to 6 µm. They are merged and branched out. The surface of the branches is rough due to particles of irregular shape and varying sizes. Notably, formations with a rose-like or half-opened bud shape can be observed, with sizes of several microns.

The membrane produced from the PLA-sucrose powder is shown in Figure 3b. The material has a round shape and a light beige color.

As preliminarily determined, single- and double-layer structures do not provide sufficient integrity for the membrane when fixed in the membrane module. However, a three-layer structure is sufficient to make the membrane mechanically durable. For this reason, three layers were printed (see Figure 1b). Thus, the membrane morphology resembles a sandwich structure, with sheet thicknesses of approximately 180, 80, and 120 µm (Figure 3c). This is in agreement with the membrane thickness measured with a micrometer (Table 1). At the bottom part, an outer contour can be observed, with a width of approximately 100 µm.

One surface of the membrane is rather smooth, with a groove of micron size (Figure 3d). In general, grooves are typical for the surface of products printed using the FDM technique. However, pores responsible for filtration ability may be located precisely within the grooves. The opposite side is rougher due to the deposited polymer formations, which is also typical for FDM products (Figure 3e). Based on the obtained results, the smoother surface was placed in contact with the feeding solution in the membrane module, similarly to the face side of asymmetric membranes, while the rougher surface (bottom side) was in contact with the permeate.

A chip of the membrane is shown in Figure 3f. As observed, the outer side of each sheet is coated with a dense layer. Under the coating, a porous layer composed of polymer blocks is present. It should be stressed that the coating forms spontaneously during printing. Specifically, the inner part of the sheets facilitates considerable permeate flux, whereas the outer part acts as a separating barrier.

**Figure 4:**
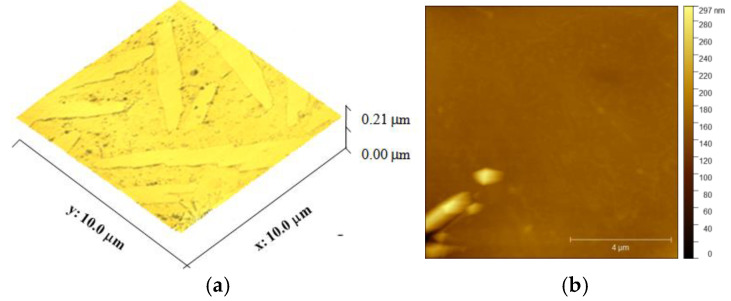
(**a**) 3D and (**b**) 2D AFM images of PLA membrane.

The two- and three-dimensional images of the face surface relief were analyzed using the AFM technique (Figure 4). The membrane surface contains elongated smooth regions, with rough regions of irregular shape in between. This roughness is caused by polymer deposition, which results in a characteristic “peak-valley” morphology [58]. At the beginning of filtration, mainly during the first 10 min, foulants accumulate in the valleys of the surface, leading to a rapid flux decrease. To quantify this effect, the mean square roughness of the surface was estimated (see Table 1). Despite the polymer deposition on the surface, which is a characteristic feature of the FDM method, the surface roughness of the PLA membrane falls within the intermediate range compared to commercial membranes. For instance, the *R_q_* value was found to be 12.5 ± 1.2 nm for a polyethersulfone membrane [59] and ranged from 10.6 ± 1.2 nm to 16 ± 3.1 nm for the same material treated with plasma [60]. Values up to 100 nm have been reported for polyamide membranes [61].

**Figure 5:**
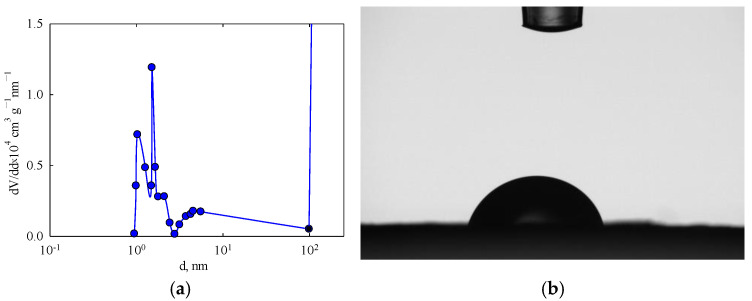
(**a**) Differential pore size distribution for the PLA membrane and (**b**) its wetting angle.

The nitrogen adsorption-desorption method revealed a weakly developed surface area and a very small pore volume, which was determined using this technique (see Table 1), namely micropores, mesopores, and macropores with sizes smaller than 50 nm. The differential pore size distribution shows several peaks (Figure 5a). Pores with a width below 3 nm are attributed to the polymer structure. Since the membrane can retain colloidal particles, the pores responsible for retention typically range between 2–100 nm [55]. More likely, larger pores correspond to the polymer blocks (see Figure 3a). The water contact angle was found to reach 69.5 ± 1.6° (Figure 5b, see also Table 1). Comparable values have been reported for filtration membranes containing polysulfone and poly (vinyl pyrrolidone) [62].

### 3.2. Water Test

**Figure 6:**
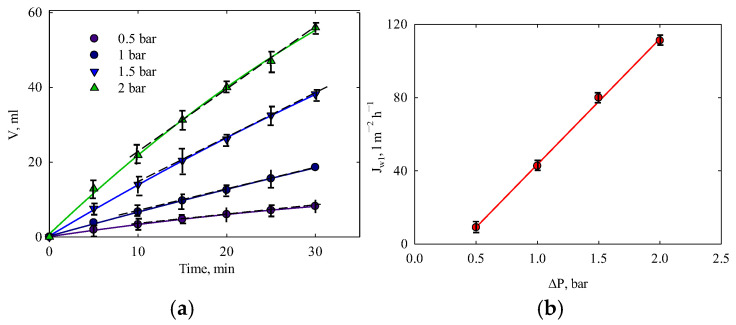
(**a**) Cumulative water volume over time of water filtration and (**b**) the effect of pressure on the permeate flux.

Unlike ion exchange polymers [63], the PLA membrane does not swell in water. However, membranes made from porous viscoelastic polymers undergo compaction under the influence of pressure [55,64]. The compression of polymer membranes leads to deformation, which reduces pore size. As a result, the permeate flux can decrease significantly, even during filtration of distilled water [55,64,65]. To study the membrane’s resistance to pressure, a water permeability test was performed. Figure 6a illustrates the cumulative volume of water passing through the membrane as a function of time. Two regions are visible: the first represents the initial slowing of water transport due to membrane compaction, while the second is linear, indicating a steady state with a constant rate of water transport. The steady state is reached 10 min after the start of filtration. For comparison, the steady-state time for an acetylcellulose membrane is approximately 1 h [65].

The water flux is proportional to pressure within the range of 0.5–2 bar (Figure 6b). In other words [55]:

(9) J=LΔP

here Δ*P* is the pressure drop and *L* is the phenomenological coefficient.

The porosity was estimated according to Equation (2). These data are given in Table 1. The porosity is lower than the values reported for certain ultrafiltration membranes (up to 0.90 for polysulfone material [66]). The acetylcellulose microfiltration membrane has a porosity of 0.62, and its modification with a rigid polymer transforms it into an ultrafiltration separator [65]. In this case, porosity decreases to 0.28–0.5.

A review of the literature shows water permeability values (L m^−2^h^−1^bar^−1^) as follows: 15–55 for a polyethersulfone microfiltration membrane modified with graphene oxide and tannic acid [67], 5–13, and 1–3 for polyacrylonitrile ultrafiltration membranes and these membranes modified with hydrated zirconium dioxide and graphene oxide, respectively [68]; and 40 for a ceramic ultrafiltration membrane [69]. Significantly higher values were reported for a commercial microfiltration membrane (800–1800 L m^−2^h^−1^bar^−1^) [70] or for a laboratory sample of polyacrylonitrile ultrafiltration membrane (about 450 L m^−2^h^−1^bar^−1^) [71]. Lower permeability water (up to 220 L m^−2^h^−1^bar^−1^) is reported for poly(amide-imide) ultrafiltration membranes [72]. Thus, in terms of porosity, the PLA membrane occupies an intermediate position among known materials. Regarding pure water permeability, the membrane is more similar to ultrafiltration separators.

### 3.3. Filtration of BSA Solution

**Figure 7:**
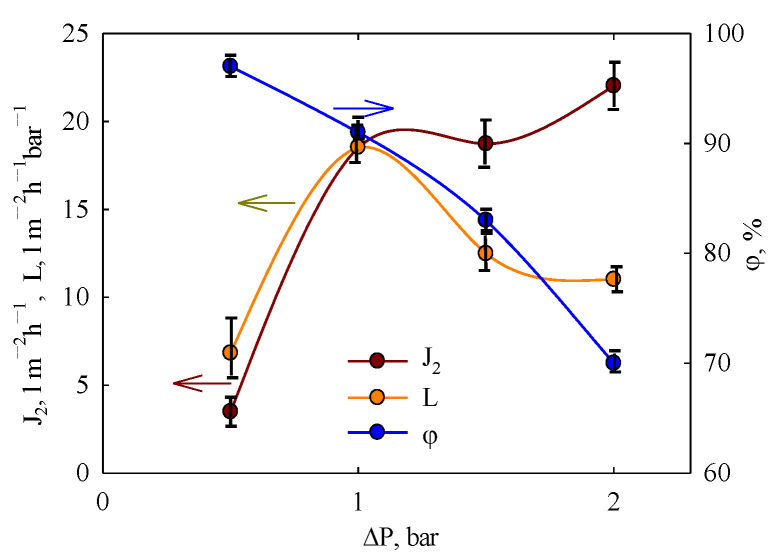
Filtration of the BSA solution: flux, permeability, and retention as functions of pressure. Arrows relate curves to ordinate axis.

Unlike water filtration, which is not affected by fouling, the *J*_2_—Δ*P* dependence is not linear when testing with a BSA solution (Figure 7). It shows a rapid increase within the range of 0.5–1.0 bar, a plateau at 1.0–1.5 bar, and is followed by a further increase. At the same time, the *L*—Δ*P* curve shows a maximum at 1 bar, indicating fouling caused by concentration polarization. This phenomenon enhances fouling, leading to a reduction in the filtration rate [55]. On the other hand, an increase in pressure enhances the permeate flux. These competing factors result in the non-linearity of the *J*_2_—Δ*P* curve and cause permeability to vary with pressure. Fouling reduces permeability by approximately five times compared to pure water (see Table 1), even under the lowest pressure. Under these conditions, the contribution of reversible and irreversible fouling to the *FDR* parameter is nearly equal.

BSA retention was estimated based on the analysis of the first portion of the permeate. The highest retention (97%) was found at Δ*P* = 0.5 bar. Thus, the membrane cut-off is estimated to be 69 kDa. As pressure increases, BSA retention declines to 70% at 2 bar, since the accumulation of species near the membrane on the feed solution side leads to their diffusion into the permeate due to the concentration gradient. This decrease in retention occurs despite of potential aggregation of protein nanoparticles under elevated pressure [55].

Thus, the combination of protein nanoparticle rejection and significant permeate flux confirms the ultrafiltration properties of the PLA membrane.

### 3.4. Orange Peel Extract

**Figure 8:**
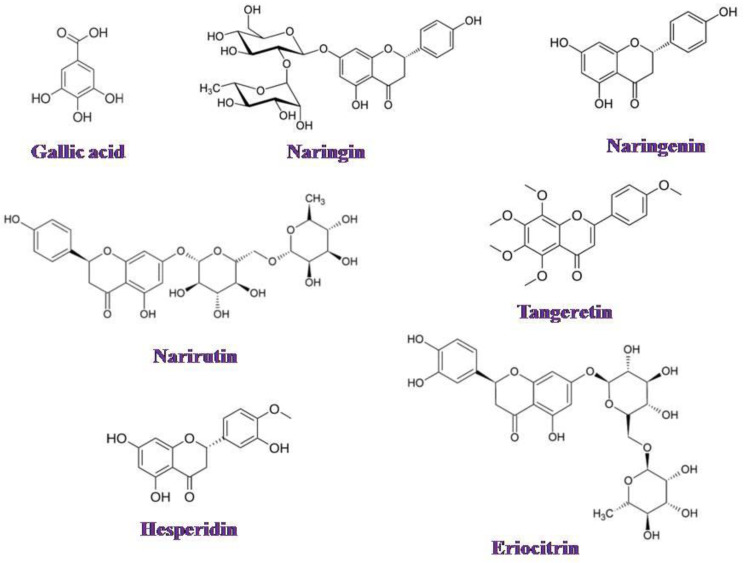
Polyphenols containing in orange peel extract (adapted from [36]).

Orange peel extracts, obtained at elevated temperatures, are yellow-colored liquids lacking aroma, as carotenoids are removed during the thermal treatment of the peel-water mixtures. Typically, these extracts contain various components that form a colloidal solution, including pectins, proteins, lipids, and polyphenols [36]. The structural formulas of these polyphenols are shown in Figure 8.

Among the polyphenols present, only gallic acid exhibits relatively high water solubility. The solubility of other polyphenols, such as naringin and naringenin, is limited to a few milligrams per liter. The polyphenol content in the extract solutions was measured as 0.33 g L^−1^ (solution I) and 1.85 g L^−1^ (solution II).

**Figure 9:**
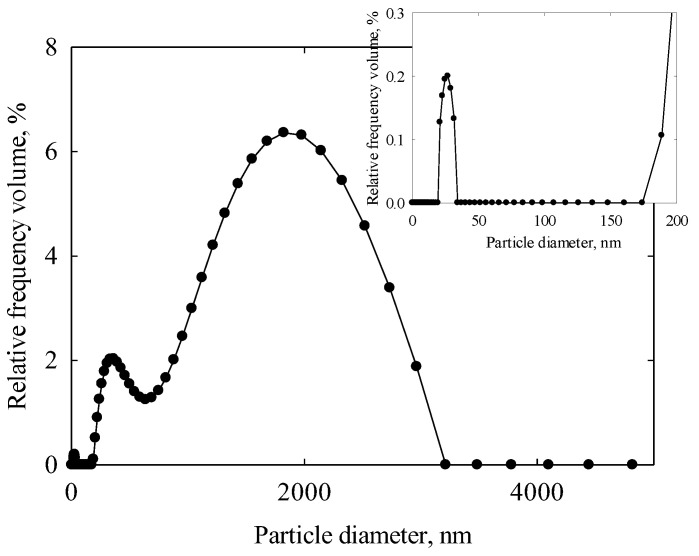
Particle size distribution in orange peel extract within the interval of 0–5000 nm. Insertion: the distribution within the diapason of 0–200 nm.

The insoluble or poorly soluble substances present in the extracts exhibit a characteristic particle size distribution, as shown in Figure 9. The curve displays three distinct peaks: 26 nm (1% contribution, insertion of Figure 9), 373 nm (22%), and 1685 nm (77%). Ultrafiltration membranes are capable of retaining particles with sizes in the range of several hundred nanometers and larger. Depending on the pore size, they may also retain smaller particles—such as those measuring 26 nm in this study–due to the sieving effect. Since the PLA membrane effectively retains BSA particles (~10 nm), it is reasonable to expect that it will also retain poorly soluble polyphenols.

### 3.5. Effect of Temperature on the Filtration of Orange Peel Extract

Temperature control is of paramount importance during filtration of biologically derived liquids to optimize separation efficiency while ensuring product quality. Generally, an increase in temperature reduces the viscosity of these liquids, enhancing their transport through the membrane [73]. However, higher temperatures also increase the diffusion coefficient of dissolved compounds, which can reduce retention due to enhanced transport from the feed solution to the permeate, driven by the concentration gradient [55]. Additionally, elevated temperatures may intensify membrane fouling by promoting the adsorption of organic substances both on the membrane surface and within its pores. Particle aggregation further exacerbates this effect, leading to a decline in filtration performance.

**Figure 10:**
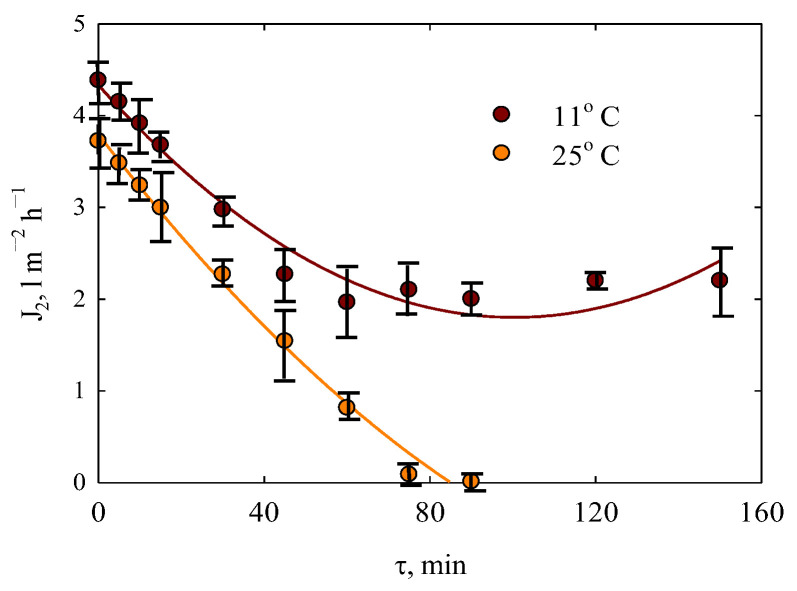
Filtration of orange peel extract (solution I, the initial volume of feeding solution was 200 mL) at 0.5 bar under different temperatures: permeate flux as a function of time.

Figure 10 illustrates the permeate flux as a function of time. To prevent significant concentration variations in the feeding solution, a large volume (200 mL) was filtered.

Under ambient conditions, the permeate flux gradually decreases, reaching zero after 1.5 h. The flux decline occurs at a rate of 2.7 L m^−2^h^−2^. At lower temperatures, the decline is slower (1.2 L m^−2^h^−2^), and a steady state is reached within approximately one hour, after which only minor fluctuations are observed. Interestingly, temperature had no significant effect on the polyphenol retention, which remained around 98% at both higher and lower temperatures. However, due to the enhanced liquid transport through the membrane at lower temperatures, filtration of orange peel extracts is recommended under these conditions.

### 3.6. Effect of Pressure and Concentration on the Filtration of Orange Peel Extract

**Figure 11:**
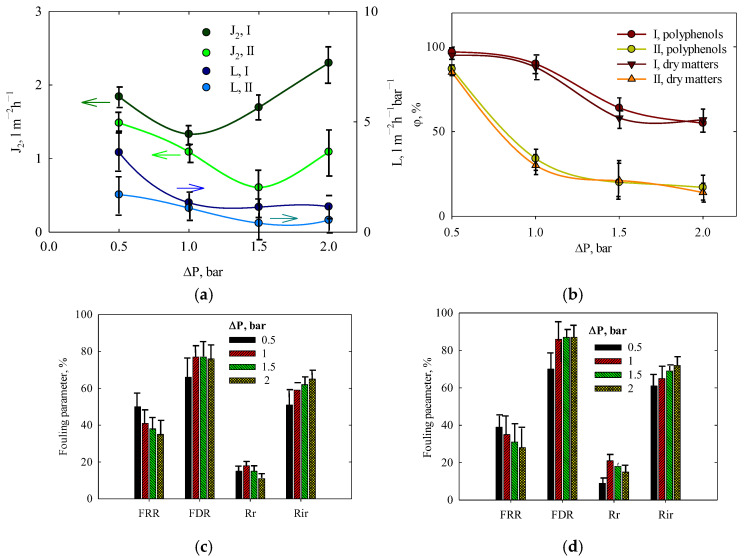
(**a**) Filtration of orange peel extract (the initial volume of feeding solution was 200 mL): permeate flux and membrane permeability, (**b**) retention against polyphenols and dry matters as functions of applied pressure. Fouling parameters obtained for solutions I (**c**) and II (**d**). Filtration was performed at 11 °C (extract) and 25 °C (water). Arrows relate curves to ordinate axis.

Unlike water filtration, which is not affected by fouling, the permeate flux of the orange peel extract gradually decreases over one hour before reaching a steady value (see Figure 10). The J_2_ values presented in Figure 11a correspond to this steady state condition, as a large volume of extract was filtered to minimize concentration variations.

The fluxes reach a minimum at 1.0 bar (solution I) and 1.5 bar (solution II) due to the interplay of two competing factors: the increase in pressure which enhances liquid transport and the intensification of fouling caused by concentration polarization which hinders filtration. A higher polyphenol content in the extract reduces the permeate flux by a factor of 1.2–3.0. However, the flux remains within the range of 0.6–2.5 L m^−2^h^−1^ at Δ*P* = 0.5–1.2 bar. A noticeable difference in permeability between solutions I and II is observed at Δ*P* = 0.5 bar, but at higher pressures, this difference becomes less significant.

The retention of polyphenols and total dry matter are given in Figure 11b. As shown, retention reaches its maximum at Δ*P* = 0.5 bar, with values of 98% and 87% for solutions I and II, respectively. Increasing pressure leads to a decline in retention, likely due to concentration polarization. The accumulation of solutes at the membrane surface on the feed side promotes their diffusion into the permeate. A higher extract concentration further intensifies the polarization. The retention of polyphenols is slightly higher than that of dry matter, as low-molecular-weight soluble compounds, such as sugars, can pass through the membrane.

Fouling parameters are shown in Figure 11c,d. As expected, an increase in pressure exacerbates fouling due to the deposition of insoluble or poorly soluble compounds, both on the membrane surface (reversible fouling) and inside pores (irreversible fouling). The *FRR* parameter is more sensitive to pressure variations, while the *FDR* value exhibits minor fluctuations. However, irreversible fouling becomes more pronounced at higher pressures. Increasing the extract concentration reduces the *FRR* parameter while increasing the *FDR* value (compare Figure 11c,d). The contribution of both reversible and irreversible fouling is greater for solution II than for solution I.

Based on these findings, the optimal pressure for extract concentration is suggested to be 0.5 bar.

### 3.7. Concentrating Orange Peel Extract

**Figure 12:**
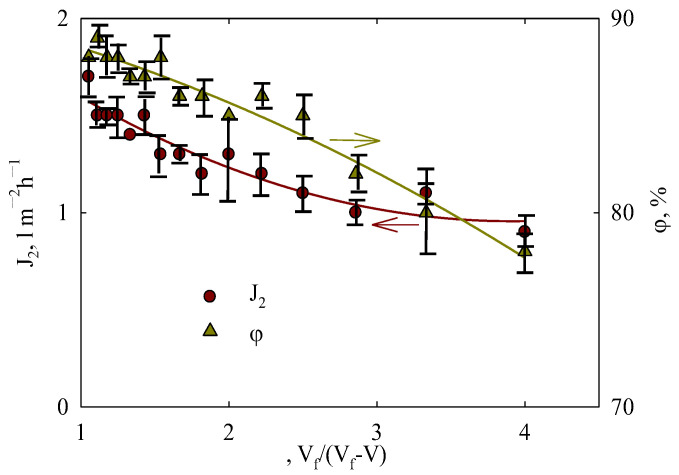
Filtration of orange peel extract (solution II. 20 mL) at 0.5 bar and 11 °C: permeate flux and polyphenol retention as functions of concentrating feed solution. Arrows relate curves to the ordinate axis.

When the concentration of solution II was performed, i.e., a small volume (20 mL) of orange peel extract (*V_f_*) was filtered, the permeate flux decreased by 11% at the beginning of separation (Figure 12). After a fourfold concentration, the filtration rate decreased by a factor of 1.9 compared with the initial value. The retention of polyphenols declined from 88% to 78%. This is evidently due to an increase in their concentration in the feed solution. The result of concentrating polyphenols is both enhanced by fouling (due to their accumulation at the membrane-solution interface) and intensified transport of species from the feed solution to the permeate as a result of the concentration gradient. Thus, it is possible to concentrate the extract by a factor of four.

### 3.8. Reproducibility of Membrane Separation

**Figure 13:**
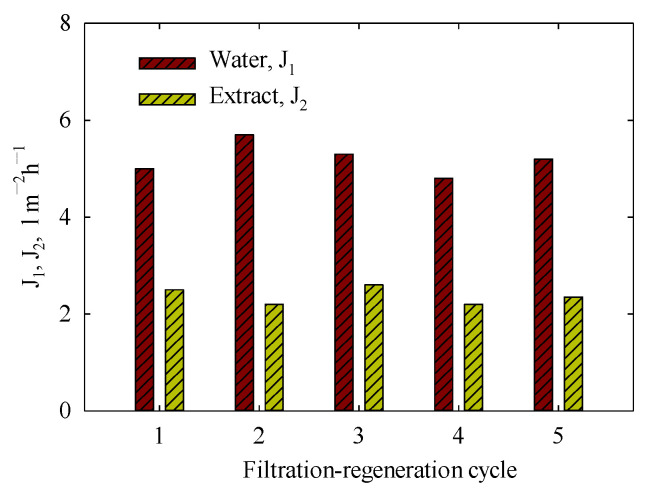
Water flux through the PLA membrane just after regeneration and permeate flux caused by the filtration of orange peel extract (solution I, the initial volume of feeding solution was 200 mL). Filtration was carried out at 0.5 bar, the temperature was 11 °C (extract) and 25 °C (water).

Figure 13 illustrates the water flux, *J*_1_, and permeate flux, *J*_2_, for several cycles of filtration of water—orange peel extract (solution I—water, followed by regeneration using an alkaline solution—acidic solution—ethanol. As seen, the rate of filtration exhibits good reproducibility: the values of both water and permeate flux show minimal fluctuations. It was determined that, *J*_1_ = 5.2 ± 0.24 L m^−2^h^−1^, *J*_2_ = 1.7 ± 0.14 L m^−2^h^−1^. The permeability values correspond to 10.4 ± 0.24 (water) and 3.4 ± 0.28 L m^−2^h^−1^bar^−1^ (extract). In all cases, the retention of polyphenols reached 97–98%. No mass losses were found after regeneration, despite the fact that PLA undergoes hydrolysis in both acidic and alkaline media [70]. This stability is attributed to the low concentration of the regenerating solution as well as the short exposition time.

According to the data of Figure 13, the membrane saves its performance at least, during 5 cycles of filtration-regeneration. Longer-term testing of membranes using solutions of different origins is a subject of further research. However, no losses of the membrane mass after regeneration allow us to suppose that the PLA membrane can be used multiple times.

### 3.9. Comparison of Obtained Results with Literature Data

According to the known literature data, filtration of polyphenol extracts is carried out at pressures ranging from 1.4 to 82 bar, depending on the type of membrane (Table 2) [47,48,74,75,76,77,78,79,80,81]. The highest permeability is observed for microfiltration membrane (Δ*P* < 2 bar), but its selectivity is rather low (up to 27%) [48]. Sufficient retention is achieved with nanofiltration, where values up to 100% have been reported [79]. However, this process requires significantly higher pressure (6–82 bar). High retention of polyphenols (up to 97%) is achieved through reverse osmosis (Δ*P* = 69 bar) [48]. Ultrafiltration has been conducted at 2–25 bar [48,74,75,76,77,78,79,80], and a maximal retention of 68% was suggested for low pressure (3–6 bar) [48]. Much higher retention (up to 95%) is pointed out in [79], however, this level is achieved at 25 bar. The lowest value (4%) was found for the UF membrane (10 kDa) [74]. However, this was a positive result, since the purpose was to separate pectins from polyphenols. Polyphenols were transported to permeate, which was further concentrated with forward osmosis. In general, it is stressed that namely ultra- and nanofiltration are the most promising methods for the recovery of polyphenols from plant extracts [82].

In our case (filtration of the solution I), nearly complete polyphenol concentrating was achieved under low pressure (Δ*P* = 0.5 bar). Regarding the filtration rate, the PLA membrane occupies an intermediate position between polymer and ceramic UF membranes (compare, for instance, [75,77]). When solution II was concentrated, the observed permeability was 2 L m^−2^h^−1^bar^−1^, and the resulting retention reached 78%. Compared to [48], the PLA membrane exhibits lower permeability but higher selectivity, which is achieved under reduced pressure.

In our case, a steady state of filtration is achieved in 40 min (see Figure 10) similar to [48] (20–40 min). Longer time was reported for nanofiltration of pomegranate juice (about 3 h) [75] and ultrafiltration of the extract of wine lees (about 1 h) [76,77]. However, no sufficient effect of pressure on the retention of polyphenols with ultrafiltration membranes has been found [75]. Nevertheless, the retention of total suspended solids increases with pressure. An increase in the permeate flux due to pressure depresses polyphenol retention over ultrafiltration [76]. Similar regularity has been found in this work (see Figure 11b). Alternately, pressure enhances the retention ability of ultrafiltration membranes [79]. Similarly, nanofiltration membranes show the improvement of retention, when pressure increases [76,79,81]. Within the pressure diapason of 5–25 bar, no change in the permeability of the nanofiltration membrane has been reported [81]. An integrated membrane process that involves ultra-, nanofiltration, and reverse osmosis has been developed [76].

As opposed to [77], a decrease in temperature improves the rate of ultrafiltration in our work evidently due to lower fouling (see Figure 10). This is probably caused by the different behavior of the organic components of the extracts of wine lees [77] and orange peel.

Extracts obtained from flowers are also a focus of attention since they contain considerable amounts of polyphenols. For instance, these compounds were recovered using ultrafiltration membranes of 1 and 10 kDa [80]. The membranes show rather high permeability, but the retention degree is not too sufficient. However, it is much higher compared with that of the UF membrane (10 kDa, [74]). The ambiguous effect of the concentrating feeding solution on the retention of polyphenols is noted in [80] as opposite to the data of Figure 12. Concentrating can depress the retention due to the diffusion of polyphenols to permeate (membrane of 1 kDa), or increase it (10 kDa) evidently due to the membrane fouling, which acts as an additional barrier against these substances. Thus, the behavior of the PLA membrane is similar to that of the membrane of 1 kDa.

Based on the analysis of literature data, it is possible to conclude that the advantage of 3D printed PLA membrane over commercial samples is the high retention of polyphenols at very low pressure. In order to achieve higher permeability and lower fouling, hydrophilization of the membrane is necessary. A possible way is a pretreatment of the PLA powder before printing (or adding a hydrophilic modifier). Another direction is a posttreatment of the printed membrane.

## 4. Conclusions

In this work, a PLA membrane was prepared in a single step via 3D printing using the FDM technique, employing a mixture of the polymer with a pore-forming agent (sucrose) as printer ink, followed by its removal from the final membrane. The membrane is composed of three layers; this sandwich-like structure provides enhanced mechanical stability. The membrane’s total porosity originated from the loose layer, which is coated on both sides with a dense film. Pores responsible for the rejection capability are located in the grooves between the polymer stripes within the dense layer.

The membrane exhibits a water permeability of 64 L m^−2^h^−1^bar^−1^, which is characteristic of ultrafiltration membranes, with a molecular weight cut-off of 69 kDa. The PLA membrane is suitable for concentrating polyphenols: its permeability ranges from 2–3.4 L m^−2^h^−1^bar^−1^ and its selectivity towards these compounds reaches 78–98%. It is possible to concentrate the extract by a factor of four. The membrane can be regenerated by washing with an acidic solution, ethanol, and water, demonstrating reproducibility in its separation properties. To mitigate fouling, tangential flow filtration is recommended. Another approach is hydrophilization of the PLA membrane which can be achieved either by incorporating a hydrophilizing agent into the printer ink or through post-treatment of the finished membrane.

It should also be emphasized that 3D printing via the FDM method is applicable not only to the fabrication of membranes for plate-and-frame configurations but also for spiral-wound and tubular devices.

## Figures and Tables

**Table 1:** Characteristics of 3D-printed PLA membrane.

Parameter		Value
Thickness, µm		402 ± 8
*R_q_*, nm (face side)		63.95 ± 8.14
N_2_ adsorption-desorption	pore volume, cm^3^g^−1^,	0.47
	specific surface area, m^2^g^−1^	9.2
Wetting angle, degree		69.5 ± 1.6
Water test	*ε*	48
	*L*, L m^−2^h^−1^bar^−1^	64
BSA test (0.5 bar)	*L*, L m^−2^h^−1^bar^−1^	7
	*φ*, %	97
	*FRR*, %	70
	*FDR*, %	80
	*R_r_*, %	42
	*R_ir_*, %	38

**Table 2:** Recovery of polyphenols with pressure-driven methods involving porous membranes.

Liquid/Target Product	Membrane	Δ*P*, bar	*L*, L m^−2^h^−1^bar^−1^	*φ,* %	Ref.
Orange peel extract	NF (aromatic polyamide, polysulfone, poly(piperazine-amide), polyethersulfone) 180–400 Da	6	2–3	54–95	[47]
Orange peel and Spinach by-products	MF (acetylcellulose)	1.4–1.8	≈50	7–27	[48]
	UF (polyethersulfone), 30–50 kDa	3–6	≈10–20	28–68	
	NF (aromatic polyamide, poly(piperazine-amide), sulfonamide)	41–82	≈0.4–1.8	70–88	
	RO (aromatic polyamide)	69	≈0.9–1.2	90–97	
Orange peel extract	UF (polysulfone), 10 kDa	3	≈8	4	[74]
Pomegranate juice	UF (polyethersulphone, fluoropolymer), 1–4 kDa	10	0.1–0.3	30	[75]
Winery lees extract, olive extract	UF (polyethersulphone), 30–50 KDa	7	1–4	5–45	[76]
Winery lees extract	UF (TiO_2_)	2	10	54	[77]
	NF (polyamide)	9	2	90	
Winery lees extract	UF (poly(vinylidene fluoride)	2	4	28	[78]
	NF (polyamide)	2	6	80	
Olive waste extract	UF (polyamide), 1–3.5 kDa	25	2–4	90–95	[79]
	NF (polyamide)		3-	98–100	
Rose petal extract	UF (polyacrylonitrile), 1 and 10 kDa	2–4	≈10	50	[80]
Fennel waste extract	MF (ceramics, Al_2_O_3_), 0.8 μm	1	25	2	[81]
	TS40 NF (material are not pointed)	5–25	5	90–95	
Orange peel extract	UF (PLA)	0.5	3.4	98	This work

## Data Availability

The original contributions presented in this study are included in the article. Further inquiries can be directed to the corresponding author.
